# Dental Providers’ Perspectives on Diagnosis-Driven Dentistry: Strategies to Enhance Adoption of Dental Diagnostic Terminology

**DOI:** 10.3390/ijerph14070767

**Published:** 2017-07-13

**Authors:** Enihomo Obadan-Udoh, Lisa Simon, Jini Etolue, Oluwabunmi Tokede, Joel White, Heiko Spallek, Muhammad Walji, Elsbeth Kalenderian

**Affiliations:** 1 Department of Preventive and Restorative Dental Sciences, School of Dentistry, University of California, San Francisco, 707 Parnassus Avenue, San Francisco, CA 94143, USA; Enihomo.Obadan-Udoh@ucsf.edu (E.O.-U.); Joel.White@ucsf.edu (J.W.); 2 Department of Oral Health Policy and Epidemiology, Harvard School of Dental Medicine, Boston, MA 02115, USA; Lisa_Simon@hsdm.harvard.edu (L.S.); Jini_Etolue@hsdm.harvard.edu (J.E.); Oluwabunmi_tokede@hsdm.harvard.edu (O.T.); 3 Faculty of Dentistry, The University of Sydney, Westmead, NSW 2145, Australia; heiko.spallek@sydney.edu.au; 4 Health Science Center, University of Texas at Houston, Houston, TX 77054, USA; Muhammad.f.walji@uth.tmc.edu

**Keywords:** clinical coding, dental practice management, dental records, healthcare quality, diagnostic terminology

## Abstract

The routine use of standardized diagnostic terminologies (DxTMs) in dentistry has long been the subject of academic debate. This paper discusses the strategies suggested by a group of dental stakeholders to enhance the uptake of DxTMs. Through unstructured interviewing at the ‘Toward a Diagnosis-Driven Profession’ National Conference held on 19 March 2016 in Los Angeles, CA, USA participants were asked how enthusiastic they were about implementing and consistently using DxTMs at their work. They also brainstormed on strategies to improve the widespread use of DxTMs. Their responses are summarized by recursive abstraction and presented in themes. Conference participants were very enthusiastic about using a DxTM in their place of work. Participants enumerated several strategies to make DxTMs more appealing including: the use of mandates, a value proposition for providers, communication and education, and integration with EHRs and existing systems. All groups across the dental healthcare delivery spectrum will need to work together for the success of the widespread and consistent use of DxTMs. Understanding the provider perspective is however the most critical step in achieving this goal, as they are the group who will ultimately be saddled with the critical task of ensuring DxTM use at the point of care.

## 1. Introduction

The routine use of standardized diagnostic terminologies (DxTMs) in dentistry has long been the subject of academic debate [1]. While the medical profession embraced the use of diagnostic terminology as part of a patient’s medical record over a century ago [2], most dental professionals retain the use of only procedure codes to describe patient care [3]. The widely-used Code on Dental Procedures and Nomenclature [4] (CDT Code) developed by the American Dental Association (ADA) is almost entirely based on dental treatment need rather than the diagnosis of a patient’s condition, and is primarily geared towards improving billing efficiency. As a result, over the years, medical diagnostic terminologies have evolved and benefitted from regular updates, standardization, and expansion [5]; dental diagnostic terminologies on the other hand have largely remained stagnant. Early attempts to synchronize the diagnostic language used by dentists included developing the: Hemprich [6]; Gregg and Boyd [7]; Winston-Salem [8]; Toronto [9]; and University of California, San Francisco (UCSF) Z-code [10] (later refined by Creighton University) systems. In addition, the World Health Organization’s (WHO) International Classification of Diseases to Dentistry and Stomatology (ICD [11]-DA) was developed in 1965 and the ADA’s Systematized Nomenclature of Dentistry (SNODENT [12]) also evolved as an ontology for the dental profession [13].

The lack of a widely-accepted, dentistry-specific diagnostic terminology severely impacted our profession’s ability to conduct oral disease surveillance as well as monitor the quality and appropriateness of care. In fact, the quality measures validated by the Dental Quality Alliance (DQA) is restricted to process measures based exclusively on procedure codes [14]. As health care systems increasingly focus on value-based reimbursement and quality-based outcomes evaluation, the absence of such measures in dentistry has become notable and potentially harmful to patient well-being [15]. Indeed, the importance of terminologies for the accurate documentation, communication, and tracking of patient diagnoses, management, and outcomes is not in dispute [9]. However, the slow uptake of these terminologies into daily practice by dental professionals inspired our study.

In 2009, a multi-institutional team of dental researchers and clinicians led by Elsbeth Kalenderian at Harvard developed the EZ Codes dental diagnostic terminology (later renamed the Dental Diagnostic System, DDS), which is an interface terminology that incorporated terms from pre-existing dental (Toronto/UCSF/Creighton Z codes; American Academy of Periodontology (AAP)’s periodontal diagnoses and the American Board of Endodontics (ABE)’s endodontic diagnoses) and medical (ICD-9/10; SNOMED) terminologies. The team successfully demonstrated its implementation into an electronic health record (EHR) within the academic dental setting [10,16] and complied with the Meaningful Use criteria of the American Recovery and Reinvestment Act [17] by mapping it to medical terminologies. The DDS has since been adopted by several dental institutions in the United States and Europe [18]. Recently, the ADA’s SNODENT and DDS terminologies were harmonized to create the SNODDS dental diagnostic terminology [19], which was adopted as an American National Standards Institute (ANSI) standard, Standard No. 2000. SNODDS is an interface terminology that allows for easy documentation of diagnostic information by using “pre-coordinated terms” or complete diagnoses (i.e., moderate chronic periodontitis) as such eliminating “multi-clicking” to “clicking to build a diagnosis”. Additionally, when mapped on the back-end to the ICD classification, it facilitates electronic billing to third-party payers for reimbursement. To the best of our knowledge, this is the first nation-wide attempt to standardize the diagnostic language of dentists’ and move us closer to becoming a diagnosis-centered profession.

All groups (regulators, payors, organized dentistry, etc.) across the dental healthcare delivery spectrum will need to work together for the success of the widespread and consistent use of DxTMs in dentistry. Consequently, we organized a multi-stakeholder conference as an educational and scientific forum aimed at identifying the challenges from various stakeholder perspectives with a goal of developing strategies to mitigate them. Because dental providers are the group who will ultimately be saddled with the task of ensuring DxTM use at the point of care, this paper reports on their perspective and discusses strategies to obtain widespread traction of DxTM use.

## 2. Methods

We hosted the ‘Toward a Diagnosis-Driven Profession’ Conference on 19 March 2016 in Los Angeles, CA, USA immediately following the 94th General Session of the International Association for Dental Research (IADR). The IADR meeting regularly enjoys robust attendance by members of the dental research community, clinicians, dental service organizations, health informaticians, governmental organizations, and dental professional organizations. Invitations were distributed to members of the American Dental Association (ADA), American Association of Dental Research (AADR), and IADR through the ADA and IADR electronic bulletin boards, respectively. Electronic invitations were also distributed to various stakeholders and organizations. Participants were divided into four domains: providers (non-academic), payers, electronic health record (EHR) vendors, and academic institutions. There were 82 participants in attendance.

The conference was divided into two major sessions: panel presentations (morning session) and participant-generated content (afternoon session) which consisted of breakout groups in which facilitated discussions were held within and among the various stakeholder groups. For the purpose of study, we will be discussing the strategies identified during the breakout sessions for providers from both academic and non-academic sites.

### Afternoon Break-Out Sessions

The non-academic provider breakout group consisted of 8 participants, while the academic institution provider breakout group consisted of 10 participants. All providers were dentists and/or representatives of US academic institutions. Each group had a facilitator and a scribe; a facilitator’s questionnaire developed for the event was used to guide the discussion and informed consent was obtained from all participants to record the discussions at the beginning of the session.

(a) The breakout session began with the question, “After today’s panel sessions, how enthusiastic are you about implementing and/or consistently using diagnostic terminologies in your place of work?” Participants were asked to rank their level of enthusiasm, and to vote on their level of enthusiasm of adoption of DxTMs in their place of work using a Likert five-point scale ranging from ‘not enthusiastic’ to ‘very enthusiastic’.
(b) After each group’s level of enthusiasm was determined, group leaders led a brainstorming session. For enthusiastic groups (which was all groups), the group leader used brainstorming to answer, “What are relevant strategies for implementation of diagnostic terminology?”
(c) Following brainstorming, participants in each group voted on the most important strategies for implementation by their group. In some groups, problems and suggested actions were also identified.
(d) The next task was for participants to identify relevant strategies for implementation of dental DxTMs from the perspective of providers. Participants were encouraged to share as many relevant strategies as they could think of, which was recorded for the group by the scribe. When all strategies were exhausted, there was another round of voting.
(e) Participants were asked to vote individually on what they believed were the five most important strategies to enhance DxTM adoption.
(f) Following this, each group collectively voted to select four top strategies to enhance adoption of DxTMs. This was followed by discussions to develop practical recommendations for the top four strategies. The breakout sessions lasted 90 min. All notes and audio recordings were collated, transcribed, analyzed, and summarized by the conference team moderators and BullsEye Resources, Sudbury, MA, USA.

## 3. Results

Overall, participants were enthusiastic about implementing and/or consistently using dental diagnostic terminologies (DxTMs) in their workplaces, with high levels of enthusiasm among all stakeholder groups. In brainstorming strategies (see Table 1) for implementing diagnostic terminologies, several common themes emerged as factors that would drive implementation across all breakout groups:

- Mandates: If the use of DxTMs were mandated at federal or state levels, or by payers, it would drive implementation.
- A Value Proposition for Providers: If providers saw value in using DxTMs, implementation would be propelled. Value could come in the form of incentives through higher reimbursement (positive incentives) for delivering quality outcomes. There could also be negative incentives, such as penalties, for failing to use DxTMs. Other elements of value could include increased efficiency and better data for decision-making and measurement of performance. If providers saw clear benefits and could answer the question: “what’s in it for me?”, it would drive implementation. Several groups discussed considering incentives similar to the “Meaningful Use” approach used with medical EHRs.
- Communication and Education: Communication and education are seen as needed to create awareness and convey the benefits of DxTMs.
- Integration with EHRs and Existing Systems: For providers to implement DxTMs, it must be easy and convenient, fit within their workflow, and save them time (or at least not take more time). Every group discussed the importance of a simple, intuitive user interface.

A challenge mentioned in several discussions is the causality dilemma. EHR vendors would be motivated to incorporate DxTMs if they saw demand from providers, but in the absence of demand are not making this a priority. Providers are not necessarily aware of and are not currently demanding DxTMs, and want EHR vendors to take the first step by incorporating it into EHRs.

### 3.1. Providers (Non-Academic)

Additional details for the strategies identified by the provider breakout group are described below.

#### 3.1.1. Incorporating Diagnostic Terminologies into EHRs

Providers believe that DxTMs will be adopted by providers if they are seamlessly incorporated into EHRs. Specific strategies included: improving the interoperability of EHRs to promote data sharing; developing a simple and clear user interface to encourage adoption; streamlining the workflow to ensure that it fits with the typical care delivery model.

#### 3.1.2. Mandate the Use of DxTM

Participants believed that mandatory, rather than voluntary, adoption of DxTMs would be one means to hasten DxTM utilization by providers. Such mandates may extend from payers (for example, reimbursing only for procedures with diagnostic terms to verify appropriateness of care), legislative, or regulatory agencies. Participants indicated that, within the United States, state-level mandates may gain the most traction as dental practice acts are regulated on a state-by-state basis.

#### 3.1.3. Educate Providers on the Purpose and Benefits of DxTMs

One reason providers may not or do not plan to adopt DxTMs might be that they are not aware of the purpose and benefits of DxTMs to their dental practice. Participants advocated the use of educational modules and training courses to increase provider awareness. Educational strategies may include continuing education courses for those in practice, and the development of cases that encourage evidence-based dental practice through analysis of DxTM codes. Such education may also show providers that practice improvement, and perhaps enhanced profitability, may result from these analyses. Additionally, the group believed that providing training on DxTMs for future providers while in dental school would quicken the pace of DxTM adoption as the dental workforce progresses.

#### 3.1.4. Provide Financial Incentives to Use DxTMs

Participants also indicated that financial incentives would encourage providers to adopt DxTMs. Suggestions included providing clinicians with a value proposition that would demonstrate the financial benefits of DxTM adoption, including calculations of return on investment. Payers (both public and private) could reimburse providers based on outcomes, rather than procedures, which would require the use of DxTMs. Explicit incentives, similar to those present for the adoption of meaningful use technologies in the United States, as well as tax benefits for providers who adopt a DxTM could be enacted. Conversely, participants suggested penalties (negative incentives) if providers fail to document diagnoses in a structured way in patient charts.

Additional strategies received the support of the group but were not selected as the four most promising strategies to hasten DxTM adoption. These strategies included focusing on quality of care and the potential of DxTMs to improve quality of care, advocating to make DxTMs the standard of care within dental practice, implementing a national benchmarking system that would allow providers to compare their performance to others, and securing the support of early adopters to encourage a domino effect in DxTM utilization. Providers also encouraged collaboration with medical providers to facilitate adoption.

### 3.2. Academic Institutions

The discussion focused on relevant strategies for implementing DxTMs among academic institutions. Among the many strategies identified for implementation, the three receiving the most support by far, in rank order, were:

#### 3.2.1. The Importance of Patients and Value-Based Care

Academic participants saw the use of DxTMs as having great value to patients through the delivery of better dental care. Some suggested strategies to create value for patients using DxTMs included: developing measurable outcomes to assess health improvement; having composite assessments of oral health status; having more precise patient risk assessments and risk-based individualized care; communicating patient risk assessments through the use of patient-oriented dashboards; showing lifetime trends of a person’s oral health status; increasing patient engagement through home-based, self-administered diagnostics; providing patient-centered care; showing the cost/benefit relationship of prevention and interventions; helping direct resources most appropriately to care for patients with various diseases.

#### 3.2.2. A Political Push by Institutional Leaders

One driver of implementation in academic institutions is a political push—even an institutional mandate—from leadership. However, to sustain support, this top-down push needs to be accompanied by buy-in from providers. A political push would only occur if institutional leaders see the potential cost savings and financial value of diagnostic terminology, and decide to mandate it. The promise of better health outcomes collected through the EHRs, which can potentially improve institutional ratings, and are not dependent on subjective patient-provided information, will also propel institutional leaders into action. Unique to academic institutions is the presence of researchers who can serve as advocates for the numerous benefits of DxTMs for research by demonstrating oral-systemic health connections and appropriateness of care variables.

#### 3.2.3. An Easier User Interface and Streamlined Workflow

For DxTMs to be implemented in an academic environment, it requires a simple, intuitive user-interface. Specific characteristics include: time-saving measures such as having fewer clicks; smart systems with pre-populated differential diagnoses and/or treatment code pairs; usability standards; automated processes; multi-platform EHRs that can be used across electronic devices—e.g., tablets and smartphones; smooth data transfer between providers and insurance companies; voice-enabled systems; and patient-friendly dashboards and reports.

Other strategies mentioned that would help implementation in academic institutions were: (a) mandating the use of DxTMs by insurers or federal regulatory bodies; (b) educating users on the purpose, value, and benefits of diagnostic terminologies; (c) commitment that DxTMs will not be changed constantly; (d) newly designed dental EHRs; and (e) adequate training.

## 4. Discussion

This paper summarizes the suggestions of academic and non-academic providers from across the dental community to enhance the adoption of DxTMs into the dental profession. As in medicine, DxTMs are necessary to assess treatment appropriateness, quality, and safety [20,21,22]. Moreover, the utilization of DxTMs facilitates the development of treatment planning skills, as it emphasizes the need for a diagnosis to drive treatment considerations [23]. Standardized DxTMs also enhances data sharing between providers at different institutions, and facilitates the conduction of high quality clinical research to benefit the public [21,24].

Unlike their counterparts in medicine, the majority of dentists in the United States operate in private practice settings, where they are less subject to federal or payer regulations [25]. Similarly, while medical insurance reimbursement has long been tied to the appropriate use of diagnostic terms, dental reimbursement persists in being determined only by procedure codes [26,27]. It is thus to be expected that some dental providers express confusion as to what DxTMs may entail, and its impact on practice [13].

An additional consideration is that the traditional solo private practice model is rapidly being supplanted by large group practices and dental service organizations (DSOs) that employ many dentists in one or more locations [28]. As in other health systems, this evolution may lead to an increased demand for standardized quality improvement, patient safety, and efficiency metrics through the use of DxTMs [29]. Our participants suggested that financial incentives and provider education on the advantages of DxTMs might enhance adoption. Large group dental practices and similar settings with more central leadership (for example, community health centers) may be most likely to see gains in efficiency and increased financial success as a result of these incentives, given the scale on which they operate [30]. A survey of providers in a large group dental practice found that the vast majority of those surveyed had positive attitudes towards dental DxTMs [13].

Providers from dental educational institutions recognized benefits for patients as well. DxTMs will help create a transition from procedure-driven care-to-care delivered when it is appropriate. They also see it as a way to create a smoother environment for students through such benefits as improved IT skills encompassing quicker EHR entries, use of shortcuts, and clinical decision support tools. DxTMs will furthermore benefit patients and students by highlighting the connection between dental health and general health.

Dental providers, even those in solo or small-group practices, are facing increased opportunities to adopt EHR technology, which may also facilitate DxTM utilization. As with other medical providers, dentists are eligible for ‘Meaningful Use’ financial incentives funded by the Health Information Technology for Economic and Clinical Health (HITECH) Act and some states have mandated that dentists adopt EHRs in their practice [31,32]. In fact, participants noted Meaningful Use as a potential model for financial incentives to increase DxTM use by dental providers. Conversely, standardized and easy to use dental DxTMs may enhance EHR adoption by dental practices, as the relative lack of clinical decision support, streamlined interfaces, and standardization specific to dentistry within EHRs have been identified as barriers to broader EHR adoption by dentists [33].

Even among institutions that have already adopted a DxTM, utilization rates may be affected by the perceived ease of use and utility [6]. A provider-friendly user interface, for example, the presence of scroll bars or vocabulary consistent with those commonly used by providers [34], will enhance adoption of DxTMs. This aligns with suggestions from our participants, who note that a streamlined and well-integrated DxTM interface within the EHR is an important strategy that will promote the adoption of DxTMs.

Diagnosis-driven changes will serve to better align dental practice remuneration with the medical system’s model, which is evolving away from fee-for-service in pursuit of the ‘triple aim’ of improved patient experience, population health, and reduced healthcare costs [35]. The widespread adoption of dental-specific DxTMs will almost certainly be a critical component of these efforts, especially since DxTMs used to describe dental conditions in the medical setting have been inconsistent [36]. Shifting the conventional fee-for-service model of dental reimbursement to a quality-based system necessitates DxTMs that will allow payers to assess the appropriateness of selected treatments [37]. The use of DxTMs has also facilitated the development of adverse event surveillance in the dental setting [22].

Mandates requiring DxTM adoption, such as those suggested by participants, may extend from either payers or from regulatory agencies in light of evidence of increased safety and improved patient outcomes as a result of DxTM use.

Participants recommended focusing efforts to enhance provider adoption of DxTM in part on the benefits for providers, e.g., increased efficiency and financial benefits. This is in line with suggestions that the ‘triple aim’ be expanded to a ‘quadruple aim’, incorporating provider wellbeing, which DxTMs (and EHRs) may facilitate for dental providers [38]. Ultimately the dental profession itself—including provider, patient, payer, EHR vendors, and regulatory stakeholders—will need to decide whether to make a commitment to quality and safety, or risk falling further behind their medical counterparts.

### Limitations

Although we had an overall attendance of 82 participants, one limitation of our study includes the small sample size of the two breakout groups considered for this manuscript (*n* = 18). However, the common themes identified across all breakout groups were also identified within the two groups, therefore we are confident that it is reflective of the sample population. In addition, participants in a conference on dental DxTMs are likely more enthusiastic than most providers or dental leaders about the use of DxTMs, and perhaps more comfortable with innovations in dental practice. Thus, these strategies may be more applicable to enhancing DxTM uptake for providers or institutions which are comfortable being early adopters instead of those who are rather more hesitant. While the strategies articulated by the group are similar to those utilized to incentivize other practice innovations and EHR use, they have not yet been tested for DxTM adoption, and their effectiveness remains to be seen [31]. Conversely, our sample represents those at the vanguard of dental practice with an interest in dental practice transformation. Our participants have likely put a greater amount of thought into the use of DxTM and how adoption could be enhanced than the average dental provider, who may be unaware of the potential benefits of the terminology.

## 5. Conclusions

Patient safety and quality, evolving EHR technology and regulations, and changes in reimbursement systems may all hasten the adoption of diagnostic terminologies (DxTMs) in dentistry. However, its acceptance by providers in private practices and academic institutions will be critical to expanding its use and ensuring seamless integration into patient care. Our facilitated session with dental providers and academic institutions points to several strategies to expand awareness of DxTMs and increase support. They include further education about the advantages of a DxTM, the value to patient outcomes a DxTM may bring, any potential financial incentives, and the importance of regulations from payers or regulatory agencies in promoting the adoption and expansion of DxTMs in dentistry.

## Figures and Tables

**Table 1:** Group-specific strategies.

Providers (Non-Academic)	Academic Institutions
Incorporating DxTMs into EHRs	The importance of patients and value-based care
Mandate the use of DxTMs	A political push by institutional leaders
Educate providers on the purpose and benefits of DxTMs	An easier user interface and streamlined workflow
Provide financial incentives to use DxTMs	

## Abbreviations

The following abbreviations are used in this manuscript:

DDS	Dental Diagnostic System
DSO	Dental Service Organization
DxTM	Diagnostic Terminology
EHR	Electronic Health Record
HITECH	Health Information Technology for Economic and Clinical Health Act
ICD10	International Classification of Diseases 10
